# Effect of the COVID-19 pandemic on utilization of essential health services in Iran evidence from an interrupted time series analysis

**DOI:** 10.1186/s12889-024-18537-3

**Published:** 2024-04-11

**Authors:** Mohammad Ranjbar, Seyed Masood Mousavi, Farzan Madadizadeh, Nahid Hosseini Dargani, Samaneh Iraji, Blake Angell, Yibeltal Assefa

**Affiliations:** 1 Department of Health Management and Economics, Health Policy & Management Research Center, School of Public Health, Shahid Sadoughi University of Medical Sciences, Yazd, Iran; 2 Department of Biostatistics and Epidemiology, Center for Healthcare Data Modeling, School of Public Health, Shahid Sadoughi University of Medical Sciences, Yazd, Iran; 3 Department of Health Management and Economics, Shahid Sadoughi University of Medical Sciences, Yazd, Iran; 4 Yazd Health District, Shahid Sadoughi University of Medical Sciences, Yazd, Iran; 5 Centre for Health Systems Science, the George Institute for Global Health, University of New South Wales, Sydney, Australia; 6 School of Public Health, the University of Queensland, Brisbane, Australia

**Keywords:** COVID-19 pandemic, Essential health services, Interrupted time series analysis, Utilization

## Abstract

**Background:**

The COVID-19 disrupted the provision of essential health services in numerous countries, potentially leading to outbreaks of deadly diseases. This study aims to investigate the effect of the COVID-19 pandemic on the utilization of essential health services in Iran.

**Methods:**

An analytical cross-sectional study was conducted using interrupted time series (ITS) analysis. Data about five indicators, including 'childhood vaccination, infant care, hypertension screening, diabetes screening, and breast cancer screening,' were obtained from the electronic health record System in two-time intervals: 15 months before (November 2018 to January 2020) and 15 months after (January 2020 to May 2021) the onset of the COVID-19 pandemic. The data were analyzed by utilizing ITS. In addition, a Poisson model was employed due to the usage of count data. The Durbin-Watson (DW) test was used to identify the presence of lag-1 autocorrelation in the time series data. All statistical analysis was performed using R 4.3.1 software, considering a 5% significance level.

**Results:**

The ITS analysis showed that the COVID-19 pandemic significantly affected the utilization of all essential health services (*P* < *0.0001*). The utilization of hypertension screening (RR = 0.51, *p* < 0.001), diabetes screening (RR = 0.884, *p* < 0.001), breast cancer screening (RR = 0.435, *p* < 0.001), childhood vaccination (IRR = 0.947, *p* < 0.001), and infant care (RR = 1.666, *p* < 0.001), exhibited a significant decrease in the short term following the pandemic (*P* < *0.0001*). However, the long-term trend for all service utilization, except breast cancer screening (IRR = 0.952, *p* < 0.001), demonstrated a significant increase.

**Conclusions:**

The COVID-19 pandemic affected utilization of essential health care in Iran. It is imperative to utilize this evidence to develop policies that will be translated into targeted planning and implementation to sustain provision and utilization of essential health services during public health emergencies. It is also vital to raise awareness and public knowledge regarding the consequences of interruptions in essential health services. In addition, it is important to identify the supply- and demand-side factors contributing to these disruptions.

## Background

Following the diagnosis of the first case of COVID-19 in Wuhan, China, in January 2020, the number of patients infected with the virus increased worldwide [1]. According to the World Health Organization's (WHO) report, as of April 2023, 763,740,140 people worldwide had been infected, and 6,908,554 had died from this disease [2, 3]. In response to the COVID-19 pandemic, countries around the globe emphasized the importance of implementing public health measures, including social distancing, quarantines, hand hygiene, and contact tracing, to control the incidence of the disease and reduce mortality rates [4]. Additionally, the prevalence of COVID-19 presented significant challenges to healthcare systems worldwide [5]. These healthcare systems encompass formal and informal organizations with diverse objectives, methods, resources, and responsibilities at local, regional, or global levels [6]. In critical situations, healthcare systems have two critical tasks: responding to emergency needs and ensuring the continuous provision of high-quality healthcare services. Among these services, reproductive services, vaccination, child and maternal health, and counseling are considered the most essential. These services aim to reduce the prevalence and mortality of preventable and curable diseases in children, mothers, and families [7].

The interruption in the provision of essential health services can be attributed to various factors, including lockdowns, media warnings, public health actions, fear of COVID-19, changes in hospital policies, and public health authorities' recommendations to stay at home in order to reduce infections and alleviate the burden on hospitals [3, 7]. According to the WHO, the COVID-19 pandemic disrupted the delivery of essential health services in nearly all countries [7].

Hung et al. (2022) reported that married women with higher education levels avoided seeking healthcare services due to concerns about COVID-19 infection. Hence, there is a pressing need for a responsive healthcare system and timely communication to prevent preventable deaths during critical events such as pandemics [8]. Doubova et al. (2021) demonstrated that the COVID-19 pandemic significantly impacted healthcare systems and preventive services, reducing utilization of services such as breast cancer screening, pregnancy prevention, vaccination, diabetes and hypertension counseling, and pre-pregnancy care. Notably, there was a two-thirds reduction in visits for breast screening counseling services and a one-third reduction for vaccination and diabetes and hypertension counseling services [7].

According to WHO estimates, 70% of this disruption primarily affected child vaccination services [9]. The interruption in the delivery of child vaccination services can decrease herd immunity and increase the risk of future outbreaks of infectious diseases, such as measles and polio [10, 11]. The consequences of this disruption are particularly severe in low and middle-income countries [12].

Two crucial milestones in routine childhood immunization programs within primary care are universal vaccinations at 8 weeks of age, including the hexavalent vaccine, and vaccines administered at one year, which coincides with the first dose of the DPT vaccine [13].

The results of previous studies in Colombia and Pakistan indicated that, despite WHO recommendations to continue children's vaccination programs during the COVID-19 pandemic, vaccination services decreased by 14.4% in Colombia and 65% in Pakistan [11, 12]. In 2021, Ota et al. conducted a review study evaluating the impact of the pandemic on global immunization activities. The results indicated that a higher number of unvaccinated vulnerable individuals could lead to the re-emergence of preventable diseases such as measles. Therefore, there is a need for guidelines and targeted vaccination operations during and after the pandemic, as well as extensive cooperation among stakeholders and frontline healthcare providers [9].

Additionally, the pandemic has disrupted the provision of screening services, treatment, and necessary measures for cancer patients, resulting in a poor prognosis for these individuals [14]. In a cross-sectional study, Yuan Li et al. (2021) investigated 4551 patients in Hong Kong using a survey form to collect information and facts. The study results revealed a correlation between reduced physician consultations and the fear of COVID-19, with a 10% decrease in medical consultations during the COVID-19 pandemic [15]. Another qualitative study by Akbari et al. (2021) examined the self-care status of these patients during the pandemic using semi-structured interviews. The findings indicated that patients did not maintain desirable self-care practices related to nutrition, mobility, exercise, and medication during the COVID-19 pandemic [16].

In Iran, the healthcare network, including health houses and comprehensive healthcare centers, was established to ensure equitable access to essential health services for all people in rural and urban areas. This network aims to improve public health, enhance the quality of life, and reduce health disparities across the country [17]. Following the onset of the COVID-19 pandemic in Iran, numerous plans were implemented for early diagnosis, treatment, hospitalization, and patient recovery. These measures included the establishment of a crisis team, the development of guidelines and protocols, and the screening of all households. As of April 2023, the number of confirmed COVID-19 cases in Iran reached 7,603,697, with 145,837 reported deaths [2, 18].

One of Iran's initial responses to the pandemic was the complete evacuation of certain hospitals to admit COVID-19 patients, the closure of clinics and schools, travel restrictions, and an emphasis on hand hygiene and social distancing to curb the spread of the coronavirus [19, 20]. Interrupted time series (ITS) analysis has been widely employed to assess the performance of essential health services during the COVID-19 pandemic. This method allows for integrating longitudinal data that cannot be incorporated using other approaches within a specified population and reports the results [21].

As far as we know, some studies in Iran regarding the impact of the COVID-19 pandemic on healthcare utilization have been conducted, but none of them have examined essential health services; rather, they have mostly studied utilization of hospital services during the pandemic. On the other hand, the existing challenges in the Iranian healthcare system, specific society needs, financial constraints and resources, and stimulation of the research system convinced researchers to conduct study related to people's utilization of essential health services within the Iranian healthcare system. Hence, this study seeks to assess the influence of the COVID-19 pandemic on the utilization of essential health services, with a focus on access to services like hypertension screening, diabetes screening, breast cancer screening, childhood vaccination, and infant care during the pandemic.

## Methods

This analytical cross-sectional study aimed to examine the impact of the COVID-19 pandemic on the utilization of essential health services in Yazd, Iran. Yazd, with an area of 74,650 square kilometers, is located in the central part of Iran and has a population of approximately 1,138,533. Essential health services in Yazd province are provided to the entire population through rural and urban comprehensive health centers, and the services provided to the covered population are recorded in the Electronic Health Record system (EHR). EHR, developed by the Ministry of Health, and Medical Education (MOHME) in Iran, is an electronic system designed to collect, record, and manage the health information of citizens. This comprehensive system offers policy makers, healthcare professionals and healthcare facilities access to population health information. EHR acquires information through various means, including citizen registration, reports from physicians and healthcare centers, vaccination details, and prescribed medication records. By collecting this data, the EHR assist healthcare professionals and healthcare centers in providing the best health services to population. It also enables better management of treatment histories and tracing of improvement and changes in health status. the most important essential health services currently provided at EHR include breast cancer screening, hypertension screening, diabetes screening, infant care, and childhood vaccination for which information on the utilization of these services is available in EHR. we used interrupted time series analysis (ITS) to focus on five key health service indices: breast cancer screening, hypertension screening, diabetes screening, infant care, and childhood vaccination. ITS analysis is particularly suitable for studying the utilization of essential health services before and after the COVID-19 outbreak because it allows for studying trends over time, controls for pre-existing trends, seasonality, and confounders, identifies immediate effects, such as changes following the outbreak, provides statistical rigor with segmented regression, and its commonly used for policy evaluation, making it suitable for assessing the impact of the COVID-19 outbreak on healthcare utilization.

In the context of this research, ITS analysis can reveal how the pandemic affected essential healthcare utilization, including changes in rates, patterns, and disparities. This information is crucial for healthcare planning and policy development during and after a crisis.

### Sampling and data collection

Our target population was all the registered population in EHR of Yazd province who were eligible to receive essential health services. We collected the recorded data related to all children under 18 months covered by the childhood vaccination program, all women aged 30 to 70 included in the breast cancer screening program, the population over 30 years old included in the diabetes screening program, the entire population over 18 years old included in the blood pressure screening program, and all infants under 28 days from EHR in 2023. Data was collected over a 30-month period, with 15 months before (November 2018 to January 2020) and after the start of the pandemic (January 2020 to May 2021).

### Statistical analysis

Statistical analysis involved paired t-tests to compare annual averages and ITS analysis to assess changes in the indicators' levels and trends. The ITS model included variables for immediate effects, long-term trends, and continuous effects.

To estimate the ITS model, accounting for the use of time series data, we assessed the correlation between time series observations in the response variable using the Durbin-Watson test. Subsequently, we employed the following regression model for each index:

$$\log\;\mathrm Y=\beta_{\mathit0}+\beta_{\mathit1}\text{T}+\beta_{\mathit2}\text{D}+\beta_{\mathit3}\text{P}+\mathrm\varepsilon$$

Where β_0_ is pre-intervention initial level of outcomes. T refers to the time since the start of the observation, and β_1_ represents is the time trend coefficient (the linear slope since the start of the intervention). D is the change variable in such a way that it is 0 before the intervention and 1 after the intervention. β_2_ refers to the immediate effect of the intervention, and P indicates the time since the start of the intervention. Its value was zero before the intervention. β_3_ refers to the continuous effects and the difference between the linear slopes before and after the intervention. ε is the model error. All statistical analysis was performed using R 4.3.1 software, considering a 5% significance level.

## Results

Table 1 displays essential health services indicators' mean and standard deviation before and after the COVID-19 Pandemic.

**Table 1:** Compare means of services number before and after COVID-19 pandemic

Indicator	15 months before pandemic		15 months after pandemic		P-Value^*^
	Mean	SD	Mean	SD	
**Breast cancer screening**	4587	1534	1861	1185.3	< 0.0001
**Hypertension screening**	2219	613	776	311.6	< 0.0001
**Diabetes screening**	662	234.9	319	124.7	< 0.0001
**Infant care**	60	7.4	515	423	< 0.0001
**Childhood vaccination**	10917	558	9579	796	< 0.0001

^*^The paired-samples t-test

According to the findings presented in Table 1, there was a notable decrease in the average utilization rates of breast cancer screening, hypertension screening, diabetes screening, and childhood vaccination services following the emergence of the COVID-19 pandemic (*p* < 0.0001). Conversely, there was an increase in the mean utilization of infant care services after the pandemic (*p* < 0.0001).

Before the onset of the pandemic, an average of 4,587 individuals underwent breast cancer screening. This number decreased significantly to 1,861 post-pandemic, marking a substantial reduction of 70%. Similarly, the average number of individuals utilizing hypertension screening services decreased from 2,219 before the pandemic to 776 after, representing a significant 54% decrease in utilization.

In terms of diabetes screening, the average number of individuals receiving these services dropped from 662 pre-pandemic to 313 post-pandemic, indicating a notable 47% decrease in utilization. Table 1 further indicates that the average number of children vaccinated decreased from 10,917 before the pandemic to 9,579 after, showing a significant 14% reduction.

On the contrary, there was a significant increase in the utilization of infant care services. Before the pandemic, an average of 60 infants received these services, which rose to 515 after the pandemic, reflecting an 85% increase in utilization.

Table 2 presents the interrupted time series results from Poisson modeling, illustrating the changes in the level and trend of essential health services utilization both before and after the COVID-19 pandemic.

**Table 2:** Results of Poisson's ITS model estimation

**Indicator**	**Changes**	**Coefficient **	**RR**	*p-value*
**Breast cancer screening**	initial level of outcomes (β_0_)	8.245	3808.53	< 0.0001
	linear slope since the start of the intervention (β_1_)	0.023	1.020	< 0.0001
	immediate effect of the intervention (β_2_)	-0.838	0.432	< 0.0001
	long term effect of the intervention (β_3_)	-0.049	0.952	< 0.0001
**Hypertension screening**	initial level of outcomes (β_0_)	7.960	2864.73	< 0.0001
	linear slope since the start of the intervention (β_1_)	-0.033	0.967	< 0.0001
	immediate effect of the intervention (β_2_)	-0.660	0.517	< 0.0001
	long term effect of the intervention (β_3_)	0.016	1.016	< 0.0001
**Diabetes screening**	initial level of outcomes (β_0_)	6.925	1017.39	< 0.0001
	linear slope since the start of the intervention (β_1_)	-0.057	0.944	< 0.0001
	immediate effect of the intervention (β_2_)	-0.123	0.884	< 0.0001
	long term effect of the intervention (β_3_)	0.036	1.037	< 0.0001
**Infant care**	initial level of outcomes (β_0_)	4.207	67.15	< 0.0001
	linear slope since the start of the intervention (β_1_)	-0.013	0.987	< 0.0001
	immediate effect of the intervention (β_2_)	0.511	1.666	< 0.0001
	long term effect of the intervention (β_3_)	0.182	1.199	< 0.0001
**Childhood vaccination**	initial level of outcomes (β_0_)	9.350	11498.8	< 0.0001
	linear slope since the start of the intervention (β_1_)	-0.006	0.994	< 0.0001
	immediate effect of the intervention (β_2_)	-0.054	0.947	< 0.0001
	long term effect of the intervention (β_3_)	0.003	1.003	< 0.0001

Table 2 illustrates a noteworthy increase in the utilization of breast cancer screening services prior to the COVID-19 outbreak, indicated by an incidence rate ratio (RR) of 1.02 with a *p* < 0.001. However, there was a significant decline shortly after the onset of the pandemic, with an RR of 0.435 and a *p* < 0.001. This reduction in service utilization remained a significant long-term trend, with an RR of 0.952 and a *p* < 0.001.

Moreover, the trend of hypertension screening, which had been decreasing before the pandemic (RR = 0.96, *p* < 0.001), experienced a substantial short-term decrease following the outbreak (RR = 0.51, *p* < 0.001). Nonetheless, after several months of the pandemic, there was a notable increase in the long-term trend of utilizing hypertension screening (RR = 1.016, *p* < 0.001).

Before the pandemic, there was a significant decrease in diabetes screening (RR = 0.944, *p* < 0.001), which further declined immediately after the pandemic (RR = 0.884, *p* < 0.001). However, the long-term trend of utilizing this service saw a significant increase after several months of the pandemic (RR = 1.0366, *p* < 0.001).

Similarly, childhood vaccination experienced a significant decrease both before (IRR = 0.994, *p* < 0.001) and immediately after the pandemic (IRR = 0.947, *p* < 0.001). However, the long-term trend of utilizing this service increased significantly after several months of the pandemic (IRR = 1.003, *p* < 0.001).

In terms of infant care, there was a significant decrease in utilization before the pandemic (IRR = 0.987, *p* < 0.001), and this decline continued immediately after the pandemic (IRR = 1.666, *p* < 0.001). However, the long-term trend of utilizing this service showed a significant increase after several months of the pandemic (IRR = 1.199, *p* < 0.001).

**Fig. 1:**
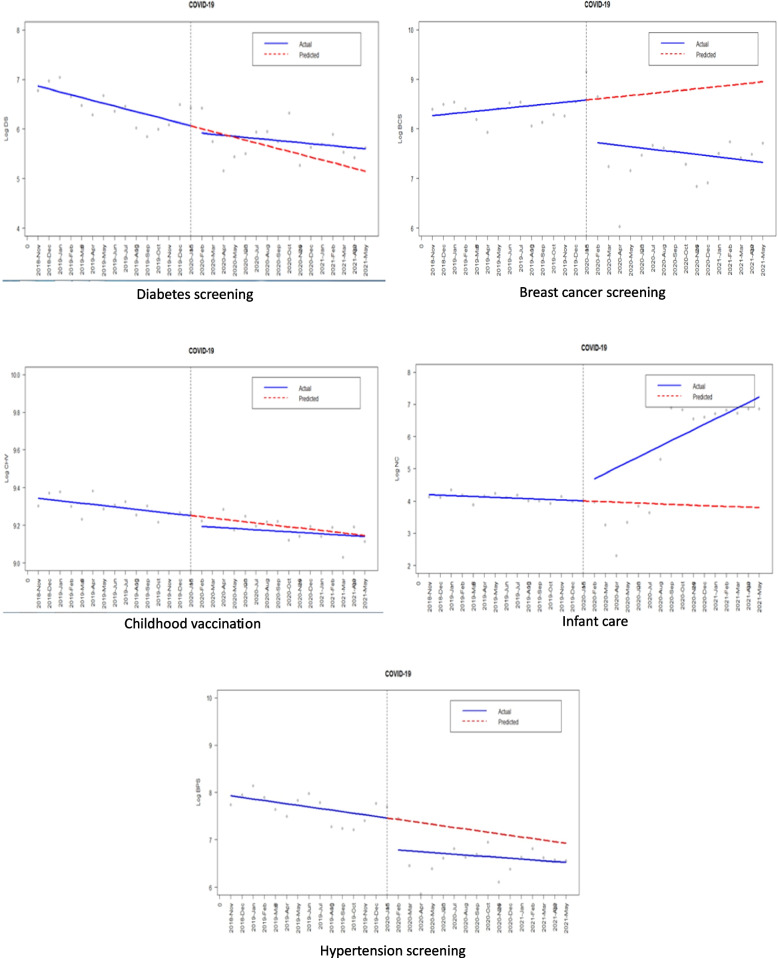
The trend of changes in selected indicators in the period before and after the covid-19 pandemic

The trend of changes in selected indicators in the period before and after the COVID-19 pandemic are depicted in Fig. 1.

## Discussion

This study investigated the profound impact of the COVID-19 pandemic on the utilization of essential health services in Yazd, Iran, employing interrupted time series analysis. This methodological approach enabled us to evaluate both immediate and enduring changes in essential health service utilization simultaneously, taking into account the prevailing conditions during the COVID-19 pandemic. Our findings unequivocally demonstrate that the utilization of all essential health services was significantly affected by the COVID-19 outbreak.

Specifically, we noted substantial declines in the utilization of breast cancer screening, hypertension management, diabetes care, and childhood vaccination services in the short term following the onset of the COVID-19 pandemic compared to the pre-pandemic period. However, it is noteworthy that there was a significant increase in the utilization of infant care services. Over the long term, the utilization of all services, except for breast cancer screening, exhibited a significant increase. Based on our estimations, 5,850 residents in Yazd were deprived of essential health services during the pandemic, with proportions ranging from approximately 70% in breast cancer screening services to about 14% in vaccination services.

A study by Doubova et al. (2021) conducted in Mexico revealed alarming figures where among nine health services, a staggering 8,074,000 people were deprived of essential services during the COVID-19 pandemic. Approximately one-third of these cases were linked to vaccination, diabetes management, hypertension screening, and infant care services [7].

The emergence of the COVID-19 pandemic had the potential to rapidly unravel significant health progress achieved over the past two decades. This impact was particularly profound in cancer screening, which suffered severely during the pandemic, leading to delayed cancer diagnoses. The management of cancer cases in primary care, spanning from diagnosis to patient treatment and follow-up, was significantly compromised during the COVID-19 pandemic [22]. We found that there was an average 70% reduction in breast cancer screening services from February 22, 2020, to May 24, 2021, compared to the corresponding period in the previous year. In practical terms, this translates to an estimated 70,914 women aged 30 to 70 being deprived of essential breast cancer screening services. the substantial decline in breast cancer screening services during the pandemic period have significant clinical implications such as: delayed cancer diagnoses, compromised outcomes, increase in advanced cases, psychological impact, and healthcare system burden. 

In the United States, a previous study reported an astonishing 94% decrease in breast and cervical cancer screening tests, with 285,000 breast cancer screening tests remaining unperformed during the COVID-19 pandemic [14, 23].

The disruption of vaccination services due to the COVID-19 pandemic is a critical issue that has received inadequate attention [24]. Globally, vaccination programs have been significantly impacted by the pandemic [25]. Building on previous research, our findings reveal a substantial 14% decrease in the utilization of vaccination services for children under 18 months following the outbreak. This decline represents one of the most pronounced reductions during the pandemic, likely stemming from parental concerns regarding potential coronavirus exposure in healthcare settings. Moreno et al. (2021) also reported a comparable decline of approximately 14.4% in vaccination coverage in Colombia from 2019 to 2020, notably affecting children under 12 months receiving the pneumococcal vaccine [12]. Furthermore, within just one week of the WHO declaring the COVID-19 outbreak a global emergency, the United States witnessed a significant decrease in vaccinations for children under 2 years of age. The results of previous studies in Pakistan indicated that, despite WHO recommendations to continue children's vaccination programs during the COVID-19 pandemic, vaccination services decreased by 65% [11]. Lina et al. (2021) highlighted a staggering 37% interruption rate in vaccination services in Saudi Arabia, primarily driven by COVID-19 fears [26]. Similarly, Doubova et al. (2021) reported a 37% reduction in vaccination services for children under 5 years old in Mexico, estimating that this led to 180,070 children missing out on vital vaccines, resulting in a 10% to 45% monthly increase in child mortality rates [7]. According to a World Health Organization report, at least 80 million children under one year old are now at risk of preventable diseases such as polio, diphtheria, and measles due to COVID-19-related disruptions in vaccination servic24).)

The era of COVID-19 has presented significant challenges for diabetic patients, impeding their ability to maintain optimal self-care. Factors such as insulin medication unavailability, increased consumption of unhealthy foods, reduced physical activity due to lockdowns, and diminished social interactions have compounded these difficulties [27, 28]. Diabetes stands out as a pivotal chronic disease, serving as a precursor to various other serious conditions such as heart disease, hypertension, vision impairments, kidney disease, and neurological disorders. Given the rising prevalence of diabetes and the tendency for many cases to remain asymptomatic for extended periods, early screening for diabetes becomes paramount [28]. Screening assumes even greater importance considering the escalating prevalence of asymptomatic diabetes cases that can lead to microvascular complications [28]. Abdi et al.'s (2020) study reveals that during the COVID-19 pandemic, a significant number of non-COVID-19 patients with pre-existing conditions like diabetes were left without necessary healthcare services. Furthermore, many individuals with diabetes experienced reduced physical activity due to global quarantines imposed by governments. These ramifications pose substantial risks, increasing the likelihood of infections, hospitalizations, amputations, and potentially fatal outcomes in diabetic patients [29]. Our study highlights a striking 47% decrease in public utilization of diabetes screening services, likely driven by pandemic-related apprehensions about visiting healthcare facilities. Consequently, remote screening methods such as phone consultations emerged as viable alternatives to traditional in-person health center visits.

Detecting hypertension promptly and managing it effectively are critical for achieving optimal control. the increasing prevalence of hypertendion is linked to more than 16 percent of the burden of coronary artery disease, 21 percent of peripheral vascular diseases, and 29 percent of strokes [30]. Our estimations indicate that approximately 54% of individuals aged 30 and above were unable to access hypertension screening services during the pandemic. Kiari et al. (2022) also reported a 10.4% reduction in hypertension screening at the outset of the pandemic [31]. Hypertension itself carries a two- to three-fold heightened risk of contracting COVID-19 or experiencing adverse outcomes, including severe infections and mortality [32].

Globally, about 16 percent of children face developmental delays, highlighting the crucial need for early identification to implement preventive interventions. Complications from prematurity stand as the primary cause of death among infants and children under 5 years old. Studies estimate that achieving 95 percent coverage of essential care for small and sick newborns could potentially save the lives of 750,000 infants by 2030 across 81 countries. A study conducted in Nepal during the COVID-19 quarantine reported a 52 percent decrease in births, a 20 percent increase in premature birth rates, and a threefold rise in infant mortality rates [33]. Additionally, studies during the coronavirus pandemic among pregnant women with COVID-19 revealed a threefold higher risk of premature birth compared to women without the disease. The escalating rates of premature births have far-reaching negative repercussions on infants, families, and healthcare systems worldwide. Small infants, especially premature ones, are particularly vulnerable to reduced healthcare coverage and diminished service quality [34].

On the other hand, concerning infant care, our study uncovered a notable increase in service utilization post-pandemic, although the exact reason for this trend remains elusive. Further investigations unveiled changes in the registration period for infant care, transitioning from 3–5 days to 3–10 days in the revised booklet distributed to health centers by the Iranian Ministry of Health and Medical Education (MOHME) in late 2020. This alteration resulted in a concurrent increase in recorded cases within the comprehensive health information system during the COVID-19 pandemic. Additionally, shifts in MOHME policies, coupled with heightened emphasis and monitoring of integrated care for healthy children in health centers during the pandemic, likely contributed to the uptick in recorded cases.

In a study by Ashish and colleagues (2020) in Nepal, the stillbirth rate rose from 14 per 1000 total births before the COVID-19 quarantine to 21 per 1000 total births, and neonatal mortality increased from 13 per 1000 live births to 40 per 1000 births [33]. Another recent study in 2022 indicates that access to and utilization of newborn care services in many countries have been impacted by reduced routine checks and preventive care for women and infants by healthcare facilities. Healthcare providers have noted a decrease in the number of women and infants using newborn care services [35]. Globally, studies suggest that around 16% of children experience developmental delays, underlining the critical importance of early diagnosis for timely preventive interventions [36].

## Limitations of the study

Our research had a few limitations. Initially, it was carried out over a 15-month period following the onset of the Covid-19 pandemic, focusing on the rapid changes in usage indicators during this brief timeframe. A longer study duration, particularly during the peak periods of Covid-19 spread, would offer policymakers a more comprehensive understanding of how a crisis impacts healthcare utilization. Secondly, being retrospective, not all data required to fully illustrate these changes were gathered, leaving room for potential unknown factors aside from Covid-19 that may have affected the outcomes. Additionally, a national study could enhance the generalizability of the findings.

## Conclusion

The COVID-19 pandemic disrupted the provision and use of health and medical services. This disruption in essential health services can lead to outbreaks of eliminated diseases and increase the mortality rate from preventable diseases. The primary drivers of this interruption are due to fear of contracting COVID-19, national quarantines, travel restrictions, limited access, and the heightened focus of managers and policymakers on COVID-19 control measures aimed at reducing its prevalence and associated mortality. It is imperative to utilize this evidence to develop policies that will be translated into targeted planning and implementation to sustain the provision and utilization of essential health services during public health emergencies. It is also vital to raise awareness and public knowledge regarding the consequences of interruptions in essential health services. In addition, it is important to identify the supply- and demand-side factors contributing to these disruptions.

## Data Availability

The data sets generated during the study are available from the corresponding author upon reasonable request.

## Abbreviations

WHO: Health Word Organization
ITS: Interrupted time series
RR: Relative risk
HER: Electronic health record
